# Pain measurement in the older people: evaluation of the psychometric properties of the Geriatric Pain Measure (GPM-24) – polish version

**DOI:** 10.1186/s12877-021-02495-1

**Published:** 2021-10-18

**Authors:** Grażyna Puto, Iwona Repka, Piotr Brzyski

**Affiliations:** 1 Department of Internal and Environmental Nursing, Institute of Nursing and Midwifery, Faculty of Health Sciences, Jagiellonian University Medical College, Kopernika 25 Street, 31-501 Krakow, Poland; 2 Department of Clinical Nursing, Institute of Nursing and Midwifery, Faculty of Health Sciences, Jagiellonian University Medical College, Kopernika 25 Street, 31-501 Krakow, Poland; 3 “Dziupla” Statistical Analyses Piotr Brzyski, Aleje Jerozolimskie 85/21, 02-001 Warsaw, Poland

**Keywords:** chronic pain, older people, psychometric properties, validity, reliability, pain assessment

## Abstract

**Background:**

Chronic pain in older people is of particular importance not only with regard to negative subjective experience but also as an indicator of the quality of medical care. Brief scales to assess pain may help health professionals with early recognition and treatment to avoid patient suffering. However, these scales should be adapted to the cultural context to provide valid assessments. The aim of this study was to evaluate the psychometric properties of the Polish translation of the Geriatric Pain Measure – 24 (GPM-24) in older people.

**Methods:**

The study was conducted among 181 people aged 65 and over with chronic (noncancer) pain of varying intensity lasting more than 6 months. Construct validity was assessed using the principal component analysis (PCA) method with oblimin rotation. Criterion validity was evaluated by correlating the scores of the GPM-24 with the scores of the McGill-Melzack questionnaire (MPQ). The reliability of the GPM-24 was estimated in terms of internal consistency using Cronbach’s alpha coefficients.

**Results:**

The PCA revealed a 6- component structure of the set of items that constituted the GPM-24. Most of these components were defined by items included in the same subscale, similar to the result obtained by the original scale’s authors. There were significant correlations between the GPM-24 and some dimensions of MPQ: affective (rho = 0.25, *p* = 0.001), present pain intensity (rho = 0.44, *p* < 0.001), pain rating index total (rho = 0.31, *p* < 0.001), and number of words chosen (rho = 0.26, *p* < 0.001). The value of the standardized Cronbach’s alpha equalled 0.89 and thus confirmed the high reliability of the GPM-24.

**Conclusions:**

The Geriatric Pain Measure − 24 is a reliable and valid tool that is recommended for the monitoring and multidimensional assessment of chronic pain in older people in daily practice as well as in clinical trials.

**Trial registration:**

Statutory research “Chronic pain in people over 65 years of age” K/ZDS/005733, conducted in 2015–2018.

**Supplementary Information:**

The online version contains supplementary material available at 10.1186/s12877-021-02495-1.

## Background

Chronic pain in older people is of particular importance not only with regard to negative subjective experience but also as an indicator of the quality of received medical care, especially if we consider the progressive ageing of society [1, 2]. There is evidence that approximately 50 % of the population of people aged 65 years and older experience pain [3, 4]. Moreover, pain incidence doubles with each decade of life [4, 5]. These findings are likely associated with the coexistence of chronic diseases in older people, which are often accompanied by chronic pain [6].

Pain may limit movement, contributing both to the loss of general physical and mental fitness [7] and to the occurrence of complications such as fall injuries [8, 9], depression, anxiety [10], and insomnia [9]. The experience of chronic pain is associated with a worse quality of life not only for the person who is suffering but also for his or her family [1, 7]. The social importance of chronic pain indicates that it causes an economic problem, but above all, it is a measure of the quality of medical care [1, 2]. Inadequate treatment of chronic pain in older people [11] remains a widespread problem that puts patients at risk of serious health consequences [1, 3, 12]. One of the components associated with inadequate pain treatment is insufficient pain assessment, which presents clinicians with unique challenges [3, 13]. Appropriate pain assessment is fundamental to optimal treatment aimed at reducing pain intensity and ultimately improving patients’ quality of life [5, 7]. To achieve more effective pain treatment in older adults, it is necessary to use population-specific standardized and validated pain assessment tools [2, 5]. An accurate, comprehensive assessment of chronic pain is a prerequisite for establishing an interdisciplinary, holistic approach [2, 5] to provide older people and their families with the most effective treatment, which can significantly improve their quality of life [1, 7].

In Poland, very few tools are available for the multidimensional assessment of pain in older people [5]. The most frequently used tools in Poland are the Visual Analogue Scale (VAS) or the Numerical Rating Scale (NRS), which are one-dimensional tools and assess only the intensity of pain [5, 14, 15]. Multidimensional scales that assess the intensity and impact of chronic pain on various aspects of functioning, such as the Brief Pain Inventory - Short Form (BPI-SF) or the McGill Pain Questionnaire (MPQ), are more complex and require much more time to answer all the questions, which is why they are more often used in younger populations [5, 14]. Due to its subjective and multidimensional nature, pain is characterized by changes in intensity and location [1, 2]. Often, especially in older people, there are no evident changes to explain its origins; tools are therefore required that assess other dimensions of pain in addition to intensity [5, 16–18]. The Geriatric Pain Measure (GPM) is among the chronic pain scales that meet the requirements for a multidimensional tool intended for the population of older people [5, 16, 19, 20]. This scale is recommended for people with multiple comorbidities [5, 16]. It assesses the intensity of pain and psychological and functional aspects, which constitute a key element of the loss of independence, decrease in fitness and exclusion from social and spiritual life [5, 16, 19, 21–24 ].

The aim of this study was to evaluate the psychometric properties of the Polish translation of the Geriatric Pain Measure – 24 (GPM-24) in older people.

## Methods

The evaluation of the psychometric properties of the GPM-24 was based on research conducted in 2015–2018. The following inclusion criteria were applied: respondents aged 65 years and older, informed consent to participate in the study, hospitalization in nonsurgical wards (e.g., rheumatology, pulmonary, laryngology, internal diseases), and patients reporting chronic noncancer pain with different degrees of severity lasting for more than 6 months. The exclusion criteria were severe cognitive deficits (0–3 points on the Abbreviated Mental Test Score - AMTS), cancer, and lack of pain (or pain lasting less than 6 months).

Initially, we recruited 305 patients who reported pain of varying severity. However, 94 (34 %) of them did not meet the above mentioned criteria after we analysed their medical records (i.e., documented cancer or cognitive impairment), and 30 (9.8 %) patients refused to participate in the study (i.e., lack of consent). 181 (59 %) older people were included in the analysis.

The study included the sociodemographic characteristics of the respondents (age, sex, education level, place of residence, marital status, financial situation, living arrangements). The assessment of cognitive functions (episodic, semantic and operational memory) was performed using AMTS [25, 26]. Functional well-being in terms of everyday activities (Personal Activities of Daily Living, P-ADL) was tested using the Katz Index of Independence in Activities of Daily Living (ADL) [27], and more complex activities were tested with Lawton’s Instrumental Activities of Daily Living (I-ADL) [28]. The severity of depression was measured using the 15-item Geriatric Depression Scale (GDS − 15) by Yesavage [29–31].

The GPM-24 was developed in English and analysed for component validity and internal consistency by Ferrell et al. [16]. The GPM-24 is a multidimensional tool for assessing pain in older people in terms of disengagement because of pain (items 9, 10, 11, 12, 15, 18, 24), pain intensity (items 13, 17, 19, 20, 21, 22, 23), pain with ambulation (items 4, 5, 6, 7), pain with strenuous activities (items 1, 2, 3) and pain with other activities (items 8, 13, 14, 15, 16). The two scale questions (items 13, 15) are both contained in two different dimensions [16, 22].

The GPM-24 includes 22 questions with binary (yes/no) answers on the negative effects of pain and 2 questions assessing pain intensity on a numerical scale of 0–10 (items 19 and 20). A total score is obtained by summing the number of “yes” answers to the pain intensity rating (0–10 points in two items) and multiplying the final score by 2.38. The final total score ranges from 0 to 100 points. A score of 0 to 29 indicates mild pain, a score of 30 to 69 indicates moderate pain, and a score greater than or equal to 70 indicates severe pain [16, 21, 24].

The Polish translation of the GPM-24 was performed by commissioning two independent certified medical interpreters to translate from English to Polish. The two obtained versions were compared in terms of content and meaning, and the necessary corrections were made to the Polish translation so that the translation reflected the authors’ intentions and the content of individual items. It was considered equally crucial to ensure that the wording of the Polish translation was correct, thus creating a natural-sounding Polish version of the questionnaire. The next step was the back translation of the newly obtained Polish version of the questionnaire into English (back translation) by two independent translators. The two retranslations were compared with the original version.

Cultural adaptation of the scale were performed after obtaining the written consent of the authors GPM-24 as part of the statutory research “Chronic pain in people over 65 years of age” K/ZDS/005733, conducted in 2015–2018, for which the approval of the Bioethics Committee Jagiellonian University KBET/83/B/2013 was obtained.

The McGill Pain Questionnaire (MPQ) is used for the quantitative and qualitative assessment of pain experience. The qualitative assessment of pain consists of adjectives describing pain in four domains: sensory (S, includes 1–10 subcategories); affective (A, with 11–15 subcategories); evaluation of pain (E, in 16 subclasses); and miscellaneous: miscellaneous sensory [M(S), 17–19] and miscellaneous affective/evaluation [M(AE), 20]. The obtained data can be presented as the number of words chosen (NWC), as the Pain Rating Index-Total (PRI-T) based on the mean values and, finally, as the indicator of pain intensity based on the Pain Rating Index-r (PRI r). Present pain intensity (PPI) is evaluated on a six-point scale so that pain can be assessed quantitatively [32]. Similarly, current pain intensity (CPI)/present pain intensity (PPI) is also assessed on a six-point scale so that pain can be assessed quantitatively [32–34].

Test-retest reliability of the scale was estimated at level at least 0.95 [35]. Factor analysis conducted by [36] provided results comparable with previous studies [37] which was accepted by the authors as proof for good construct validity of the scale.

### Statistical analysis

Qualitative variables are presented as counts (n) and percentages (%). Quantitative variables are presented as the means and standard deviations (SD).

To determine the psychometric properties of the scale, the validity was evaluated in terms of content, criterion and construct. Construct validity was assessed using the principal component analysis method (PCA) with oblimin rotation, with the delta parameter equal to zero and Kaiser normalization. As the criteria for the number of extracted components, an eigenvalue greater than 1 and the interpretation of components were used. The correlation between the extracted components and between scale items was expressed as the Pearson r coefficient.

Criterion validity was assessed by correlating the GPM-24 scores with the MPQ scores to assess the extent to which a given tool produces results consistent with other tools that are considered appropriate for measuring the same construct validity.

Construct validity was assessed by estimating the Rho Spearman rank correlation between the GPM-24 and other tools such as the AMTS [25, 38], P-ADL by Katza et al. [27], I-ADL by Lawton and Brody [28], and GDS by Yesavage [29–31], which measure domains that are perceived to be associated with pain.

The reliability of the GPM-24 was estimated in terms of internal consistency using unstandardized and standardized Cronbach’s alpha coefficients.

## Results

### Sample characteristics

In the analysed sample, the percentage of surveyed women was higher than that of men (61.9 % vs. 38.1 %). The largest group was those who were divorced or widowed (55.5 %), more than half of the respondents lived with their family (60 %), and nearly half of the respondents had secondary education (47.5 %) (Table 1).

**Table 1:** Sociodemographic characteristics of the study sample

Sociodemographic characteristics		mean	SD
Age (years)		77.1	7.9
Gender		N	%
women		112	61.9
men		69	38.1
Education		N	%
primary		20	11.0
vocational		36	20.0
secondary		86	47.5
university		39	21.5
Place of residence		N	%
urban areas		127	70.2
countryside		54	29.8
Marital status		N	%
married		81	44.8
divorced or widowed		100	55.2
Financial situation		N	%
gets by		100	55.0
hardly gets by		81	45.0
Living arrangements		N	%
living alone		36	20.0
with a partner		36	20.0
with a family		109	60.0
		mean	SD
Number of comorbidities		4.1	2.4
Geriatric Depression Scale – GDS − 15 (range 0–15)		6.7	2.8
Abbreviated Mental Test Score – AMTS (range 4–10)		8.4	1.6
Activities of Daily Living (ADL) by Katz (range 0–6)		5.0	1.4
Instrumental Activities of Daily Living (I-ADL) by Lawton (range 0–27)		19.1	4.9
McGill Pain Questionnaire (MPQ)	sensory (S 1–10)	7.8	5.9
	affective (A 11–15)	1.9	2.0
	evaluation (E 16)	2.0	1.4
	miscellaneous sensory (M/S 17–19)	1.9	1.8
	miscellaneous affective/evaluation (M(AE) 20)	1.2	1.2
	Present Pain Intensity (PPI)	3.3	0.6
	Number of Words Chosen (NWC)	7.7	3.9
	Pain Rating Index-Total (PRI-T)	17.91	8.6
	Pain Rating Index-r (PRI r)	2.4	0.5

N - number of respondents; % - percentage of respondents; SD – standard deviation; GDS − 15: 1–15 points severe depression; 6–10 points moderate depression; 0–5 points no depression; AMTS: 0–3 points severe cognitive impairment; 4–6 points moderate cognitive impairment; 7–10 points normal condition; ADL by Katz: 0–2 points severe functional impairment; 3–4 points moderately disabled persons; 5–6 points, the activity is fully preserved; I-ADL by Lawton: The more points, the better the efficiency (complex)

### Validity

The PCA applied to the set of variables that constituted the Polish version of GPM-24 extracted 7 components with eigenvalues greater than 1, which jointly explained 64.9 % of the total variance of this set of items. However, the interpretation of the extracted components for this solution and for those with different numbers of components led to the choice of a 6- component solution. It explained 60.6 % of the total variance and provided better reproducibility of the original component structure of the GPM-24 scale. For the first component, its highest component loadings had 6 variables, including 3 from the pain with ambulation subscale, 2 from the pain with other activities subscale and 1 from disengagement. The second component was defined by 4 items, including 3 that in the original scale constituted the pain with strenuous activities subscale and 1 item from the pain with ambulation subscale. For the third component, the 6 items from the disengagement subscale and 1 from pain with other activities had the highest component loadings. The fourth component was defined by 1 item from pain with other activities subscale and 2 items from the pain intensity subscale, and the fifth component was defined by another item from the same subscale. For the sixth component, the 4 items from the pain intensity subscale and 1 item from the pain with other activities subscale had the highest component loadings. There were 4 items that cross-loaded on 2 components: items 6 and 15 for the 1st and 2nd components, item 14 for the 2nd and 6th components, and item 17 for the 3rd and 4th components (Table 2). As the rotation method that was used allowed for nonorthogonal components, the highest correlations were assessed between the 1st and 6th components (r = -0.43), the 3rd and 6th components (r = -0.36), and the 1st and 3rd components (r = 0.33), whereas the other correlations between components did not exceed 0.2.

**Table 2:** Matrix of rotated principal components of variables included in the GPM-24 scale

Item	Subscale	Component					
		1	2	3	4	5	6
	**Disengagement because of pain**						
9	Limited time for work or other activities			0.53		0.35	
10	Achieving less than expected			0.48			
11	Restriction of the type of work or other activities			0.70			
12	Need to make an extra effort at work or during other activities			0.57			
15	Lack of enjoyment of social gatherings or other recreational activities	0.38	0.32				
18	Relying on family members or friends for help because of pain			0.72			
24	Feelings of sadness or depression			0.73			
	**Pain intensity**						
13	Trouble sleeping				0.82		
17	Feeling tired physically or mentally			0.38	0.42		
19	Intensity of pain that is usually experienced						− 0.60
20	Average pain intensity level over the last 7 days						− 0.81
21	Never completely disappears						− 0.68
22	Experienced every day						− 0.67
23	Experienced a few times a week					0.89	
	**Pain with ambulation**						
4	Walking up the stairs more than one floor		− 0.52				
5	Walking up the stairs more than a few steps	0.83					
6	Walking further than one block	0.51	− 0.45				
7	Walking one block or a shorter distance	0.85					
	**Pain with strenuous activities**						
1	Vigorous movement		− 0.83				
2	Limiting strenuous activities		− 0.77				
3	Lifting/carrying grocery shopping		− 0.45				
	**Pain with other activities**						
8	Bathing or getting dressed	0.80					
14	Inability to participate in religious services		0.31				− 0.34
16	Lack of possibility to travel or use standard means of transport			0.39			

From the PCA table, all the factor loadings below 0.4 were removed for a clear presentation of the factor structure of the analysed scale

The reliability of the total score was estimated at 0.85, whereas the value of the standardized Cronbach’s alpha coefficient was estimated at 0.89. The mean correlation between scale items and their total score equals 0.45 and ranges from 0.12 to 0.65.

The reliability of the subscales of the Polish version, as proposed by the scale authors, was satisfactory: two subscales were characterized by reliability below 0.7, whereas the lowest reliability score was very close to 0.6 (Table 3). Less satisfactory results were obtained in terms of items whose removal caused an increase in the value of the reliability coefficient. Such items were found in three subscales: the removal of item 23 in the pain intensity subscale increased the alpha from 0.57 to 0.58, the removal of item 13 from the pain with other activities subscale increased its reliability from 0.63 to 0.65, and the removal of item 3 from the pain with strenuous activities subscale increased its reliability from 0.77 to 0.81.

**Table 3:** Reliability of the subscales based on the components extracted by the authors of the scale

Subscale	Cronbach’s alpha (standardized)	Item-scale correlation
disengagement because of pain	0.76 (0.77)	0.49 (0.38–0.60)
pain intensity	0.57 (0.61)	0.34 (0.09–0.54)
pain with ambulation	0.83 (0.83)	0.66 (0.56–0.74)
pain with strenuous activities	0.77 (0.80)	0.64 (0.56–0.70)
pain with other activities	0.62 (0.61)	0.38 (0.18–0.56)

Regarding the correlations between the GPM-24 and the MPQ scale, there were a number of positive correlations, starting from the highest: PPI, PRI (T), number of words chosen (NWC), affective, sensory, miscellaneous affective/evaluation (M(AE) 20), pain rating index r- (PRI r). Positive correlations were also obtained for the GPM-24 and age as well as GDS, whereas the negative correlations concerned the GPM-24 and AMTS, ADL by Katz, and the Lawton scale (Table 4). The GPM-24 score was higher in women than in men (Me = 44.1. Q1 = 33.3, Q3 = 50.0 vs. Me = 40.5. Q1 = 26.2, Q3 = 47.6, *p* = 0.014)

**Table 4:** Construct and criterion validity of the GPM-24

Characteristics		GPM-24
		rho
Age		0.18^*^
Geriatric Depression Scale (GDS − 15)		0.45^***^
Abbreviated Mental Test Score (AMTS)		-0.40^***^
ADL by Katz		-0.45^***^
I-ADL by Lawton		-0.54^***^
McGill Pain Questionnaire (MPQ)	sensory (S 1–10)	0.14
	affective (A 11–15)	0.25***
	evaluation (E 16)	− 0.03
	miscellaneous sensory (M/S 17–19)	− 0.05
	miscellaneous affective/evaluation (M(AE) 20)	0.12
	Present Pain Intensity (PPI)	0.44***
	Number of Words Chosen (NWC)	0.26***
	Pain Rating Index-Total (PRI (T))	0.31***
	Pain Rating Index r- (PRI r)	0.1

*p < 0.05; **p < 0.01; ***p < 0.001; p-value - for Spearman’s rho correlation coefficient

## Discussion

The study presents the results of the first adaptations of the GPM-24 scale to Polish cultural conditions. We showed that the Polish version of the scale had similar validity and reliability as other adaptation of GPM-24 known to authors of this paper [16]. Principal component analysis conducted on data collected from a sample of Polish people aged 65 years and older with chronic (noncancerous) pain showed the presence of 6 components that explained 61 % of the total variance in the scale variables. A review of other studies conducted in 3 European countries, Great Britain, Germany and Switzerland, showed that 5-factor structures were obtained in exploratory factor analyses, explaining 59 % (in Great Britain and Switzerland) and 62 % (in Germany) of the total variance of the analysed variables [21]. In studies on the cultural adaptation of the Turkish version of the GPM-24, the PCA distinguished 5 components with eigenvalues > 1, explaining 63 % of the total variance [19]. The authors of the Portuguese version did not report the results of the factor analysis [22], just as the authors of the original scale Principal component analysis showed the existence of 7 components with eigenvalues greater than > 1 in the tested sample, but the solution with 6 components allowed for more accurate interpretation of the components and thus better reflected the structure of the scale. However, it can be concluded that the set of variables in Polish cultural conditions has a 4- component structure because two components of PCA solution were defined by only one variable with a high component loading (component 4 defined by question 13; component 5 by question 23), while one of them was additionally defined by one variable with component loadings with similar values ​​for two components (question 17: components 3 and 4). The obtained component structure largely corresponds to the structure obtained by the authors of the original scale [16], however precise comparisons are not possible because the authors do not provide the result of PCA. Regarding the components defined by more than 2 variables, most of the variables came from the same subscale as defined by the authors of the scale: 3 out of 4 variables from the “pain with ambulation” subscale defined the first component, while all variables from the “pain with strenuous activities” subscale were among the variables that defined the second component of the PCA solution. 6 among 7 variables calculated based on data collected with the Polish version of the GPM-24. Variables from the “disengagement because of pain” subscale were mostly among the variables defining the third component, while the variables from the “pain intensity” subscale in the Polish sample were defined by two components: 2 of the 7 variables from this subscale had the highest component loadings on the fourth component, while 4 variables were defined by the sixth component. The only subscale that behaved completely differently in the Polish sample than in the original version was the “pain with other activities” subscale. Variables from this subscale had their highest component loadings on 4 out of 6 identified components. Moreover, as far as this subscale is concerned only questions about pain associated with bathing or getting dressed and questions about problems with sleeping had their highest component loadings on a level higher than 0.4, which allowed for their unambiguous assignment to a given component. On the other hand, 2 of the remaining variables (participation in religious services and social gatherings) had component loadings of similar value on two components. The reasons for these results might be rooted in the experience of chronic pain in older people, which limits their participation in religious services and causes their withdrawal from social meetings, recreational activities, travelling, and standard means of transport

The obtained results of the factor analysis are consistent with the results of a study conducted in the three aforementioned European countries, where the assessment of the 24 variables forming the GPM-24 scale, showed that 17 items exactly followed the pattern that was hypothesized a priori by Ferrell et al. [16, 21]. The results of this study also showed that the variables from the subscale of “strenuous activities” were characterized by the largest number of differences in belonging to the particular factors extracted by the PCA. The authors of this study suggest that the extracted factors are related to concepts of disengagement because of pain, pain intensity, pain with ambulation, the affective component of pain, and pain with other activities [21]. In the Turkish research, the authors also indicated several variables that loaded on a factor different than the one reported by the authors of the original scale; these were questions 7, 8, 13, 15, 17, 18, 21 and 24 [19]

The authors of the Polish study also noticed deviations from the original factor component. According to the authors of the scale [16], variables 13 and 15 had loadings of similar value on more than one component. However, in contrast to the results presented by the authors of the scale, who found only 2 such variables, the Polish version included 4 such variables (i.e., questions 6, 14, 15 and 17). Across all three Western European versions, five items cross-loaded onto more than one subscale, and six items failed to load onto any subscale. The authors of the Polish study also noticed deviations from the original factor structure. In Great Britain, items 2, 6 and 23 had the highest loadings on 2 factors, while in the German sample, items 20 and 7 had the highest loadings. In the Swiss sample, no high-ranking items were found for two factors, similar to the Turkish sample.

The authors of the scale did not find any variables that could not be classified into any of the subscales because of excessively low values for component loadings [16], however they did not present values of component loadings which could be used for comparison of the component structures by the authors of adaptations of the GPM-24. Based on the PCA results in the Polish sample, it can be concluded that the highest component loadings of several variables (3, 10, 14, 15, 16 and 17) were still too low (< 0.5) to define any of the components. Loadings that are excessively low ( < = 0.45 according to the authors of this article) to include in any of the subscales were also found in a study conducted in Great Britain (3, 23, 8), Germany (8) and Switzerland (2, 18 and 24) [2].

The reliability of the scale total score obtained in the Polish study was lower than that obtained by the authors: the value of the standardized Cronbach’s alpha coefficient was 0.89 compared to 0.94 [16]. Lower reliability of the total score than that obtained by the authors was also obtained in the studies conducted in 3 European countries, Great Britain, Germany and Switzerland (std alpha = 0.91 for each country) [21]. In Turkey, the reliability equalled 0.85 [19], while in Portugal, it ranged from 0.73 to 0.79 [22]. The authors of the scale did not report the reliability of each subscale separately, so it is not possible to compare the internal consistency of the subscales with the original scale. However, the standardized values ​​calculated in the Polish sample for 3 of the defined subscales (disengagement, ambulation and strenuous activities) were satisfactory, while for the remaining two subscales, acceptable values ​​of Cronbach’s alpha coefficients were obtained - the lowest alfa equalled 0.57 for pain intensity. It must be noticed, however, that in other adaptations of GPM-24 there also exist subscales for which reliability is below the threshold of 0.7 usually treated as satisfactory for group comparisons [23]. Such a situation may indicate a common issue which causes the process of adaptation of GPM-24 to other cultures demanding special attention. In studies conducted in four European countries, the reliability for individual subscales ranged as follows: for the subscale “disengagement because of pain”, the reliability was 0.80 in the Swiss sample, 0.81 in the German sample, 0.83 in the British sample, and 0.93 in the Turkish sample, whereas in our study in Poland, it equalled 0.76; for the “pain intensity” subscale, the reliability equalled 0.73 in the British sample and ranged from 0.79 to 0.83 for the remaining samples, whereas in Poland, it equalled 0.57. The reliability for the “pain with ambulation” subscale ranged from 0.80 to 0.83 (0.83 in Poland). The reliability for the subscale of “pain with strenuous activities” equalled 0.59 in the Swiss sample, 0.64 in the British sample, 0.70 in the Turkish sample, and 0.77 in Poland. For the “pain with other activities” subscale, the reliability equalled 0.66 in the Swiss sample, 0.67 in the Turkish sample, 0.71 in the British sample, 0.75 in the German sample, and 0.62 in Poland [19, 21]. The observed differences in the reliability of the scale and its subscales are a consequence of differences in its factor structure, and usually concern similar subscales in several countries.

The analysis of the correlation between the GPM-24 and other scales in the Polish study, as in the study conducted by Ferella, showed a significant correlation between the GPM-24 scale and the MPQ in terms of PPI, NWC, and PRI-T [16]. Moreover, the study by the authors of the scale showed a correlation between the GPM-24 and the sensory subscale, while the Polish study showed a correlation between the GPM-24 and the affective subscale. In the Polish study, as in the study by the authors of the scale, higher GPM-24 results were confirmed in women than in men, which confirms the theory that women are more prone to pain than men [38].

A positive correlation coefficient was also obtained for the GPM-24 score with age and GDS, while a negative correlation was found for AMTS, Katz’s ADL, and Lawton’s IADL scale. In the study by the authors of the original scale, correlations were confirmed only between the GPM-24 and medical problems [16].

Differences in the factor structure may be caused by differences in the characteristics of the studied sample. In the Polish sample, the average age of the respondents was 77.1 years (± 7.9), in Western Europe, it was 74.4 years (± 6.0), while the authors of the scale validated it in an older population whose average age was 84.7 years (± 6.0).

The Polish sample consisted of people hospitalized in nonsurgical wards (e.g., rheumatology, pulmonary, laryngology, internal diseases) with various types of chronic, noncancer pain lasting more than 6 months, while the original GPM-24 was developed and tested in community-dwelling older patients of outpatient clinics in Western European countries. In the study conducted in three European countries, the mean number of chronic diseases was 2.8 (± 1.7); it was 4.1 (± 2.4) in the Polish study, while it was 7.6 (± 6.0) [16, 21] in the study by the authors of the scale. These patients may suffer from acute pain related to conditions which caused hospitalization, which may impact component structure of the scale, which is built on items with dichotomous answer scale, strictly reflecting presence or absence of the acute pain. In particular, the existence of acute pain may have caused lack of clear component structure observed in case of pain with other activities subscale, where depending on different conditions acute pain may influence different fields of everyday activities.

One of the main reasons for the reported differences in the factor structure of the scale may be that it consists of questions with binary (yes/no) answers. Most of the scale questions seem to measure hidden variables that are continuous. Assessing such variables on a binary scale requires the use of a cut-off point, which may differ individually within the same population and may also be conditioned by cultural components that differ for different populations. Older people who experience mild chronic pain can answer both yes and no when asked whether they experience any pain. This is probably caused by the fact that with age-associated involution, changes alter the understanding of certain concepts, and the way one looks at oneself and pain ailments changes. Unfortunately, there are still many stereotypes in society about chronic pain in older people that have not been confirmed by scientific research. Older people often avoid pain assessments and do not inform health care professionals about experienced pain, believing that it is a natural phenomenon at their age that one simply needs to accept and that quiet suffering leads to a “tolerance” towards it. Moreover, older people often believe that reporting pain might lead to the need for diagnostic tests and hospitalization.

The study has certain limitations. The group of respondents in our study had different characteristics than the groups described in the referenced studies, which may explain the differences in the validation study validation results. Hence, culture-related pain perception by both older persons and health professionals may have an impact on the evaluation of the psychometric properties of the GPM-24 scale. It is not possible to compare precisely the validity and reliability of the Polish version of the scale with the assessment conducted for the original scale, as the authors did not include the result of PCA in their paper nor did they estimate the reliability for extracted subscales [16]. This should be considered in the appropriate interpretation of its results. Nevertheless, our research has some strengths. The study is the first in Poland to conduct a psychometric analysis of the GPM-24 scale among 181 respondents who were aged 65 years and older. The study used additional tools, such as the AMTS, GDS-15, ADL by Katz and I-ADL by Lawton, unlike other researchers in different countries.

## Conclusions

Despite the observed differences in the validity and reliability between the Polish version and the original version of the scale, the Polish Geriatric Pain Measure-24 scale is a reliable and valid tool that is recommended for the multidimensional assessment and monitoring of chronic pain in older people in everyday practice and clinical trials. We strongly recommend its use and further testing of its psychometric properties to confirm its validity in different subpopulations of older people.

## Supplementary information

**Additional file 1**

## Data Availability

The datasets used and/or analysed during the current study are available from the corresponding author [GP] on reasonable request.

## Abbreviations

GPM–24: Geriatric Pain Measure – 24
PCA: principal component analysis
VAS: Visual Analogue Scale
NRS: Numerical Rating Scale
BPI-SF: Brief Pain Inventory - Short Form
MPQ: McGill Pain Questionnaire
AMTS: Abbreviated Mental Test Score
P-ADL: Personal Activities of Daily Living
ADL: Activities of Daily Living
I-ADL: Instrumental Activities of Daily Living
GDS: Geriatric Depression Scale
S: sensory
A: affective
E: evaluation
M(S): miscellaneous sensory
M(AE): miscellaneous affective/evaluation
NWC: number of words chosen
PRI-T: Pain Rating Index-Total
PRI r: Pain Rating Index-r
PPI: Present Pain Intensity
CPI: Current Pain Intensity
SD: standard deviations
