# Supplementary material for: Pain measurement in the older people: evaluation of the psychometric properties of the Geriatric Pain Measure (GPM-24) – polish version
Source: BMC Geriatr. 2021 Oct 18;21:560. doi: 10.1186/s12877-021-02495-1 (PMC8522084; doi:10.1186/s12877-021-02495-1)
Supplement: Supplementary file 1 — Additional file 1 [file 12877_2021_2495_MOESM1_ESM.docx]

**Pain measurement in the older people: evaluation of the psychometric properties of the Geriatric Pain Measure (GPM-24) – Polish version**

Grażyna Puto^1^, Iwona Repka^2^, Piotr Brzyski^3^

^1^Department of Internal and Environmental Nursing, Institute of Nursing and Midwifery, Faculty of Health Sciences, Jagiellonian University Medical College, Kopernika 25 Street,

31-501 Krakow, Poland

^2^Department of Clinical Nursing, Institute of Nursing and Midwifery, Faculty of Health Sciences, Jagiellonian University Medical College, Kopernika 25 Street,

31-501 Krakow, Poland

^3^"Dziupla" Statistical Analyses Piotr Brzyski, Aleje Jerozolimskie 85/21, 02-001 Warsaw, Poland

**Supplementary Information**

**Geriatryczna skala pomiaru bólu (Polish Version)**

Nazwisko: Data:

| Proszę udzielić odpowiedzi na każde z poniższych pytań: | Odpowiedź | | Punkty |
| --- | --- | --- | --- |
| 1. Czy odczuwasz/odczuwałbyś/odczuwałabyś ból w trakcie energicznego ruchu np. bieganie, podnoszenie ciężkich przedmiotów lub podczas uprawiania sportów wysiłkowych? | Tak | Nie |  |
| 1. Czy odczuwasz/odczuwałbyś/odczuwałabyś ból wykonując umiarkowanie ciężkie czynności np. przesuwanie ciężkiego stołu, przesuwanie odkurzacza, granie w kręgle czy golfa? | Tak | Nie |  |
| 1. Czy odczuwasz/odczuwałbyś/odczuwałabyś ból podnosząc lub niosąc zakupy spożywcze? | Tak | Nie |  |
| 1. Czy odczuwasz/odczuwałbyś/odczuwałabyś ból wchodząc na więcej niż jedno piętro? | Tak | Nie |  |
| 1. Czy odczuwasz/odczuwałbyś/odczuwałabyś ból wchodząc już po kilku schodach? | Tak | Nie |  |
| 6. Czy odczuwasz/odczuwałbyś/odczuwałabyś ból pokonując piechotą odległość większą niż od jednej przecznicy do następnej? | Tak | Nie |  |
| 7. Czy odczuwasz/odczuwałbyś/odczuwałabyś ból pokonując piechotą odległość od jednej przecznicy do następnej lub krótszy odcinek? | Tak | Nie |  |
| 8.Czy doświadczasz bólu gdy kąpiesz się lub ubierasz? | Tak | Nie |  |
| 9. Czy z powodu bólu ograniczyłeś/ograniczyłaś czas spędzany na pracy lub wykonywaniu innych czynności? | Tak | Nie |  |
| 10. Czy osiągasz mniej niż od siebie oczekujesz z powodu bólu? | Tak | Nie |  |
| 11. Czy ograniczyłeś/ograniczyłaś rodzaj pracy lub innych czynności z powodu bólu? | Tak | Nie |  |
| 12. Czy praca, którą wykonujesz bądź inne czynności wymagają dodatkowego wysiłku z powodu bólu? | Tak | Nie |  |
| 13. Czy masz problemy ze spaniem z powodu bólu? | Tak | Nie |  |
| 14. Czy ból powoduje, że nie możesz uczestniczyć w nabożeństwach religijnych? | Tak | Nie |  |
| 15. Czy ból powoduje, że nie możesz cieszyć się ze spotkań towarzyskich lub innych aktywności rekreacyjnych (innych niż nabożeństwa religijne)? | Tak | Nie |  |
| 16. Czy ból powoduje/powodowałby, że nie możesz podróżować lub korzystać ze standardowych środków transportu? | Tak | Nie |  |
| 17. Czy ból powoduje, że czujesz się znużony/znużona fizycznie lub psychicznie? | Tak | Nie |  |
| 18. Czy z powodu bólu zmuszony/zmuszona jesteś do zależności od członków rodziny lub znajomych? | Tak | Nie |  |
| 19. Na skali od 0 do 10, gdzie 0 oznacza brak bólu a 10 – najgorszy ból jaki sobie możesz wyobrazić, określ jaki ból dzisiaj doświadczasz?  0 1 2 3 4 5 6 7 8 9 10 ………/10 |  |  |  |
| 20, Na skali od 0 do 10, gdzie 0 oznacza brak bólu a 10 – najgorszy ból jaki sobie możesz wyobrazić, określ średni poziom bólu, którego doświadczałeś/doświadczyłaś przez ostatnie 7 dni?  0 1 2 3 4 5 6 7 8 9 10 ………/10 |  |  |  |
| 21. Czy doświadczasz bólu, który nigdy całkowicie nie znika? | Tak | Nie |  |
| 22. Czy codziennie odczuwasz ból? | Tak | Nie |  |
| 23. Czy odczuwasz ból kilka razy w tygodniu? | Tak | Nie |  |
| 24. Czy w ciągu ostatnich 7 dni ból powodował, że odczuwałeś/odczuwałaś smutek lub przygnębienie? | Tak | Nie |  |
| Punktacja: Każda odpowiedź "TAK” otrzymuje 1 punkt. Punkty należy zsumować.  WYNIK CAŁKOWITY (0-42):………. WYNIK SKORYGOWAN (Wynik całkowity x 2.38) (0-100):…………  <30 łagodny ból 30-69 umiarkowany ból >70 silny ból | | | |
